# Validation of a Textile-Based Wearable Measuring Electrocardiogram and Breathing Frequency for Sleep Apnea Monitoring

**DOI:** 10.3390/s24196229

**Published:** 2024-09-26

**Authors:** Florent Baty, Dragan Cvetkovic, Maximilian Boesch, Frederik Bauer, Neusa R. Adão Martins, René M. Rossi, Otto D. Schoch, Simon Annaheim, Martin H. Brutsche

**Affiliations:** 1 Lung Center, Cantonal Hospital St. Gallen, Rorschacherstrasse 95, 9007 St. Gallen, Switzerland; dragan.cvetkovic@unibas.ch (D.C.); maximilian.boesch@kssg.ch (M.B.); otto.schoch@kssg.ch (O.D.S.); martin.brutsche@kssg.ch (M.H.B.); 2 Faculty of Medicine, University of Basel, Petersplatz 1, 4001 Basel, Switzerland; 3 Empa, Laboratory for Biomimetic Membranes and Textiles, Lerchenfeldstrasse 5, 9014 St. Gallen, Switzerland; ba.frederik@gmail.com (F.B.); neusa.martins@empa.ch (N.R.A.M.); rene.rossi@empa.ch (R.M.R.); simon.annaheim@empa.ch (S.A.)

**Keywords:** sleep apnea, polysomnography, multi-sensor, wearable, monitoring belt

## Abstract

Sleep apnea (SA) is a prevalent disorder characterized by recurrent events of nocturnal apnea. Polysomnography (PSG) represents the gold standard for SA diagnosis. This laboratory-based procedure is complex and costly, and less cumbersome wearable devices have been proposed for SA detection and monitoring. A novel textile multi-sensor monitoring belt recording electrocardiogram (ECG) and breathing frequency (BF) measured by thorax excursion was developed and tested in a sleep laboratory for validation purposes. The aim of the current study was to evaluate the diagnostic performance of ECG-derived heart rate variability and BF-derived breathing rate variability and their combination for the detection of sleep apnea in a population of patients with a suspicion of SA. Fifty-one patients with a suspicion of SA were recruited in the sleep laboratory of the Cantonal Hospital St. Gallen. Patients were equipped with the monitoring belt and underwent a single overnight laboratory-based PSG. In addition, some patients further tested the monitoring belt at home. The ECG and BF signals from the belt were compared to PSG signals using the Bland-Altman methodology. Heart rate and breathing rate variability analyses were performed. Features derived from these analyses were used to build a support vector machine (SVM) classifier for the prediction of SA severity. Model performance was assessed using receiver operating characteristics (ROC) curves. Patients included 35 males and 16 females with a median age of 49 years (range: 21 to 65) and a median apnea-hypopnea index (AHI) of 33 (IQR: 16 to 58). Belt-derived data provided ECG and BF signals with a low bias and in good agreement with PSG-derived signals. The combined ECG and BF signals improved the classification accuracy for SA (area under the ROC curve: 0.98; sensitivity and specificity greater than 90%) compared to single parameter classification based on either ECG or BF alone. This novel wearable device combining ECG and BF provided accurate signals in good agreement with the gold standard PSG. Due to its unobtrusive nature, it is potentially interesting for multi-night assessments and home-based patient follow-up.

## 1. Introduction

Sleep apnea (SA) is the most common sleep-related breathing disorder characterized by repetitive pauses or shallow breathing during sleep. SA is categorized into either obstructive SA (OSA) or central SA (CSA), or a combination of both (mixed SA). OSA is the most common form of SA and occurs by relaxing the tongue and soft palate repetitively during sleep, which causes the upper airway to narrow or completely close, limiting airflow and breathing [1]. SA is a prevalent condition, and recent population-based studies estimate its prevalence from 14% to 50% in men and from 5% to 23% in women [2,3]. Non-treated SA is associated with an increased risk of cardiovascular disease, neurocognitive impairment, reduced productivity at work, and car accidents [4].

Single-night polysomnography (PSG) is the clinical gold standard for the diagnosis of SA [5]. This laboratory-based procedure is expensive and relatively complex. It may also disturb sleep for various reasons, including sleeping in unfamiliar surroundings [6]. Furthermore, a significant inter-night variability in the measurable severity of SA has been reported [1], which may impact clinical decision making.

Portable sleep-monitoring devices have been increasingly developed in the last decade [7,8,9]. These devices require a high sensitivity of the diagnostic method in order to ensure a low rate of false negative tests [10]. The recent developments of algorithms opened doors for new approaches for the assessment of SA severity [11]. Although the use of multi-parameter approaches in selected patient populations is generally recommended [12], several algorithms have been developed for the detection of SA from electrocardiogram (ECG) recordings alone [13,14]. In this context, a novel wearable ECG acquisition system has been developed by the Swiss Federal Laboratories for Materials Science and Technology (Empa) [15,16]. The continuous multi-night monitoring of ECG signals by the portable system in a home setting was made possible due to its specific design, including porous textile ECG electrodes and a humidification unit. In a first study, the validity of this ECG acquisition system was evaluated in patients with SA by comparing the textile ECG electrodes (RR intervals) to the ones obtained with patched ECG during PSG. Data showed high levels of agreement between ECG measurements acquired from PSG and the wearable system [11]. The prediction accuracy of SA severity reached an acceptable rate of 74% (sensitivity: 88%; specificity: 61%).

As part of a follow-up project, the aim of the current study was to evaluate the clinical added value of overnight ECG monitoring combined with recordings of excursion-derived breathing frequency (BF) to predict SA severity using a wearable belt in a population of patients with suspicion of SA. An agreement analysis between the belt and the PSG data was performed. The feasibility of ECG and BF measurements with the wearable belt was also evaluated in a home setting.

## 2. Materials and Methods

### 2.1. Patients

Fifty-one patients with a suspicion of SA were included in the study. For diagnostic purposes, they were referred for a whole-night PSG investigation to the sleep laboratory of the Cantonal Hospital St. Gallen and were additionally equipped with a multi-sensor wearable monitoring belt for a single-night investigation. In addition, some patients agreed to test the device at home for a few nights.

The study was performed in accordance with the Declaration of Helsinki, following the principles of good clinical practice. The study was approved by the local institutional review board (EKOS 19/038), and all patients gave their written informed consent to participate in the study.

### 2.2. Textile Multi-Sensor Belt

**Figure 1:**
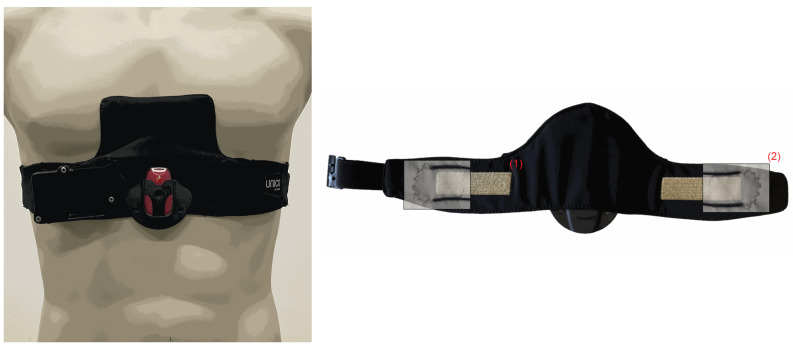
Textile multi-sensor belt for continuous monitoring of cardiac and breathing parameters. The left panel displays the belt mounted on the thorax. The right panel displays the skin-facing site of the multi-sensor belt with embroidered electrodes (1) and pressure-sensitive optical fibers (2).

A textile multi-sensor belt for combined monitoring of cardiac and breathing-related parameters, including ECG and BF, was developed by Empa (Figure 1) [15,16]. The ECG electrodes were directly incorporated into the semi-elastic polyester belt (Unico Swiss Tex GmbH, Alpnachstad, Switzerland) by embroidering of Ag/Ti-coated polyester yarn (Serge Ferrari Tersuisse AG, Emmenbrücke, Switzerland) and combined with a wetting pad for continuous humidification of the electrodes (Unico Swiss Tex GmbH, Alpnachstad, Switzerland) [17]. The ECG signal was acquired by a commercial data logger (Faros 180, Bittium Biosignals Ltd., Kuppio, Finland). A pressure-sensitive optical fibre was integrated at the edges of the monitoring belt in-between the two fabric layers. The flexible and sensing fiber (thermoplastic mono-component Geniomer fiber) was connected to a coated polydimethysiloxane fiber used for light transmission with low loss of light [18]. This fiber was connected with the light source (light-emitting diode) and the photodiode to measure changes in light intensity due to strain added to the Genomier fiber. Photodiode and light-emitting diode were components of a custom-made data acquisition system.

### 2.3. Heart Rate and Breathing Rate Variability Analysis

ECG signals were extracted simultaneously from the belt and PSG. An ECG signal quality algorithm was applied to assess the plausibility of the R-peaks detected. The algorithm considered 15 s segments of ECG signals and calculated Pearson’s correlation coefficient (*r*) for QRS and PQRST complexes. The details of the algorithm will be provided in a manuscript currently in preparation. It can be summarized as follows: The single complexes were compared to the reference complex of the respective segment calculated as median from all the complexes included in the segment. A stable and good-quality ECG signal results in a high correlation coefficient, while high variation in the morphology of the complexes reduces the correlation coefficient. Based on preliminary data, an r>0.98 was required for acceptable accuracy of QRS or PQRST complexes within the time segment. If the accuracy requirement was not met, physiological plausibility of the RR intervals detected within a time segment was assumed if they were within a range of 333 ms and 2000 ms (referring to a heart rate of 180 and 30 beats per minute, respectively), and individual RR intervals deviated not more than 33% from the median value of a specific time segment.

Heart rate variability (HRV) analysis was performed on whole-night quality filtered ECG signals. HRV analysis was subdivided into time-domain and frequency-domain analyses. Short segments of RR time series with a window width of 300 s were specified. Short-time Fourier transforms were used to calculate the power bands of the heart rate signal. Following the implementation of the R package RHRV [19], six features were derived from the time-domain analysis including:

- Standard deviation of the NN intervals (SDNN)
- Proportion of interval differences of successive RR intervals greater than 50 ms (pNN50)
- Root mean square of successive differences (rMSSD)
- Inter-quartile range of the RR time series (IRRR)
- Median of the absolute values of the RR time series (MADRR)
- Heart rate variability triangular index (HRVi), corresponding to the integral of the density distribution (number of all RR intervals) divided by the maximum of the density distribution

The following five features derived from the frequency domain were included in the analysis:

- Ultra low frequency (ULF) (band: 0–0.003 Hz)
- Very low frequency (VLF) (band: 0.003–0.03 Hz)
- Low frequency (LF) (band: 0.03–0.15 Hz)
- High frequency (HF) (band: 0.15–0.4 Hz)
- Ratio of low frequency over high frequency (LFHF)

Certain frequency bands tend to correlate with activity of the parasympathetic nervous system [20].

In analogy to the HRV analysis, breathing rate variability (BRV) analysis (see e.g., [21]) was applied to the whole-night BF recordings derived from the belt (acquired from thoracic excursion) and the PSG (acquired from impedance pneumography from the thorax and abdomen). Peaks for thorax and abdomen excursion signals were detected, and individual breathing cycles (templates) were compared to the respective reference template for time segments of 32-second duration. The segment-specific reference templates were adjusted for total breathing cycle duration, duration of inhalation phase, and amplitude of excursion. Based on preliminary data analysis (manuscript in preparation), a minimum of r=0.75 was required for an acceptable accuracy. In addition, breath-to-breath intervals shorter than 5 or longer than 50 breaths per minute were excluded from the analysis. The same time-domain and frequency-domain analysis features used in HRV analysis were also used for the BRV analysis, simply interchanging the ECG signal with the BF signal.

### 2.4. Statistical Methodology

Bland-Altman agreement analyses [22] were performed to compare data obtained from the belt and the PSG. For this purpose, whole-night mean instantaneous heart rate (HR) and mean instantaneous breathing rate (BR) were compared between belt- and PSG-derived signals in every patient with both available measurements.

Two-class support vector machines (SVM) were used to assess the classification accuracy of SA severity based on AHI. Different cut-off values defined by quantiles of AHI were investigated, and the number of true positives (TP), true negatives (TN), false positives (FP), and false negatives (FN) was calculated. The sensitivity (TP/(TP+FN)) and specificity (TN/(TN+FP)) of the HRV/BRV-based classifiers were calculated. Receiver operating characteristic (ROC) curves were used for graphical representations. The area under the ROC curves and associated 95% confidence intervals were provided as an indicator of the performance of the classifiers. Furthermore, a leave-one-out cross-validation (LOOCV) procedure was performed in order to minimize bias in the AUC estimates and avoid the risk of overfitting. In LOOCV, each observation is successively withdrawn from the original data set, and the statistic is computed on the remaining samples. This resampling technique is generally computationally efficient and particularly appropriate for small data sets.

The within-patient reliability of multiple measurements in a home setting was assessed using repeatability estimation in the framework of mixed effects modeling as implemented in the R package rptR.

All analyses were done using the R statistical software (v. 4.4.0) [23], including the extension packages RHRV [19], rptR [24], and drc [25].

## 3. Results

### 3.1. Patients Characteristics

**Figure 2:**
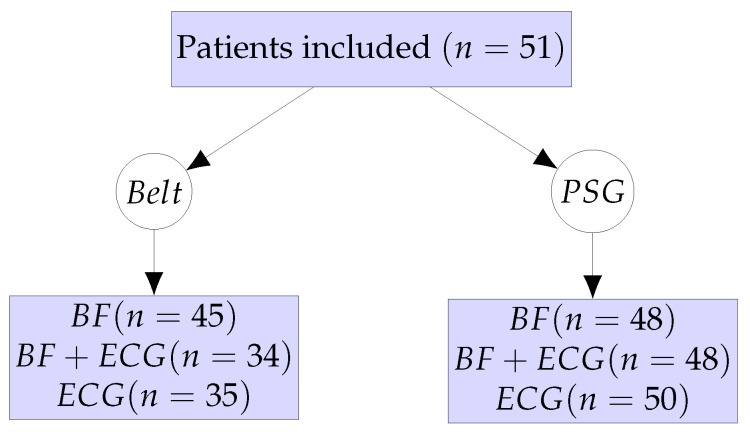
Patient workflow and numbers of eligible data for sub-analyses.

In-hospital PSG investigations were performed on 51 patients concomitantly wearing the monitoring belt. The patient workflow and the different sub-analyses are summarized in Figure 2. After quality control of the signal of the monitoring belt, BF data from 45 patients and ECG data from 35 patients could be finally analyzed. From the PSG examinations, BF data from 48 patients and ECG data from 50 patients could be analyzed. About one third of ECG measurements and 12% of excursion-derived BF measurements from the monitoring belt were of unsuitable quality, whereas 2% and 6% of ECG and BF measurements from the PSG were of unsuitable quality, respectively.

**Figure 3:**
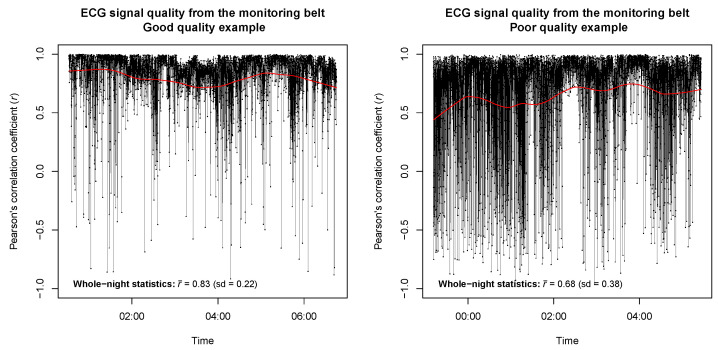
Whole-night ECG signal quality of the monitoring belt. Two illustrative patients are presented, including a good quality data set (**left panel**) and a poor quality data set (**right panel**). A locally-weighted polynomial regression smoother was applied and is represented by a red line. The whole-night statistics measured by the whole-night averaged Pearson’s correlation coefficient ($\bar{r}$) and associated standard deviation are provided at the bottom left corner of both panels.

Two illustrative exemples of whole-night ECG signal quality derived from the monitoring belt in patients with good quality and poor quality signals are represented in Figure 3.

The patient characteristics of the 51 patients are summarized in Table 1.

Sixty-nine percent of the patients were males. The median age was 49 years, and the median body mass index (BMI) was 31 kg/m^2^. Patients’ SA severity was characterized based on PSG observations by a median apnea-hypopnea index (AHI) of 33 events per hour and a median oxygen desaturation index (ODI) of 21 desaturations per hour. Moreover, patients had a median Epworth sleepiness scale (ESS) score of 9. The vast majority of patients (86%) had obstructive SA, among which three patients (6%) were diagnosed with mixed SA. No apnea events were detected in 7 patients (14%).

### 3.2. Agreement of ECG and BF Signals from PSG and Belt

**Figure 4:**
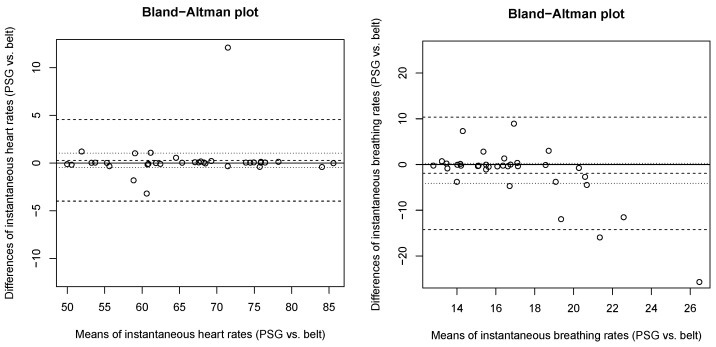
Bland-Altman agreement analyses comparing whole-night mean instantaneous heart rates (HR, **left panel**) and mean instantaneous breathing rates (BR, **right panel**) measured by the belt and PSG. The mean difference (bias) and 95% limits of agreement are represented by dashed lines. The 95% confidence intervals of the mean difference are represented by dotted lines.

The observed agreement between the mean instantaneous heart rates and mean instantaneous breathing rates derived from belt and PSG is summarized in Figure 4. As regards instantaneous heart rates (Figure 4, left panel), a non-significant bias of 0.29 beat per minute (95% CI: −0.46 to 1.04) was found with limits of agreement ranging from −3.99 (lower limit) to 4.57 (upper limit). Regarding instantaneous breathing rates (Figure 4, right panel), a non-significant bias of −1.93 breaths per minute (95% CI: −4.12 to 0.25) was found with limits of agreement ranging from −14.22 (lower limit) to 10.36 (upper limit). A higher discrepancy between the belt and PSG was observed in higher breathing frequencies.

### 3.3. Prediction of SA Severity

**Figure 5:**
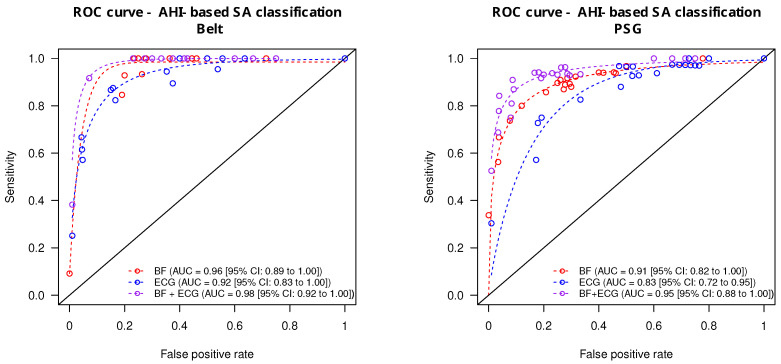
ROC curves of the prediction accuracy of apnea severity from the monitoring belt (**left panel**) and the PSG (**right panel**). The area under the curves (AUC) obtained from ECG (blue circles), BF (red circles), and the combined ECG + BF (purple circles) are provided together with the 95% confidence intervals. Smoothing ROC curves are represented by dashed lines.

The prediction accuracy of the ECG and BF signals taken alone or combined (ECG + BF) derived from the monitoring belt and the PSG is summarized in Figure 5. From the monitoring belt (Figure 5, left panel), the combined ECG + BF signals provided the highest accuracy for the prediction of apnea severity (AUC = 0.98 [0.92 to 1.00]). The AUC from the ECG signal alone was significantly lower (AUC = 0.92 [0.83 to 1.00]; p<0.033) whereas the BF signal alone provided an intermediate prediction accuracy (AUC = 0.96 [0.89–1.00]). From the PSG-derived signal (Figure 5, right panel), the prediction accuracy of the combined ECG + BF was also the highest (AUC = 0.95 [0.88 to 1.00]). The ECG signal alone provided a lower prediction accuracy (AUC = 0.86 [0.72 to 0.95]), whereas the BF signal alone provided an intermediate diagnostic accuracy (AUC = 0.91 [0.82–1.00]).

Using a leave-one-out cross-validation procedure, the sleep apnea classification accuracy provided an AUC*_loo_* = 0.97 [0.95 to 0.97] for the combined ECG + BF signals, an AUC*_loo_* = 0.94 [0.92 to 0.99] for the BF signal alone, and an AUC*_loo_* = 0.92 [0.92 to 0.93] for the ECG signal alone.

### 3.4. Multiple-Night Home-Based Assessment

In order to test the signal quality of the monitoring belt in a home setting, part of the study population agreed to test the device for multiple night home measurements. Thirty-four patients agreed to test the monitoring belt at home for up to 4 nights. Overall, 77 nightly measurements were obtained in a home setting. From the night measurements, 68 (88%) were of sufficient quality regarding the ECG-signal and 39 (51%) regarding the BF signal.

The median intra-individual repeatability was 78% (IQR: 68 to 91%) for the ECG measurements and 71% (IQR: 40 to 89%) for the BF measurements. The inter-night variability was noticeably higher on patients with severe SA compared to patients with mild SA.

## 4. Discussion

In the current study, we evaluated the diagnostic and clinical value of nocturnal ECG and BF measurements acquired from a textile-based multi-sensor monitoring belt and compared them to signals acquired during laboratory-based PSG. Overall, a diagnostic accuracy of 92% (sensitivity: 0.92, specificity: 0.93, AUC: 0.98) was achieved for the classification of SA severity with the combined ECG and BF signals derived from the belt. This was significantly higher than single parameter classifications based on either ECG or BF signals alone. In a previous study using a one-lead ECG sensor [11], we concluded that the ECG belt provided signals comparable to patched ECG, which could be used for the assessment of SA severity. However, we saw the potential to improve the diagnostic precision of the device by adding an additional BF sensor. Indeed, the addition of a BF sensor improved the prediction accuracy of SA severity.

According to the American Academy of Sleep Medicine (AASM), which used a 4-tier approach for the classification of portable monitoring systems—type 1 and 2: full attended and unattended polysomnography; type 3: unattended outpatient and inpatient polygraphy; type 4: 1 or 2 channel monitoring—our diagnostic device including two channels corresponds to a type 4 portable monitor [12].

Limitations of the current study include some reliability issues experienced with the monitoring belt. This was particularly noticeable regarding the ECG sensors, where about one-third of the measurements needed to be excluded. As an extension of the current study, some patients were offered to test the belt at home. A total of 77 home-based night ECG measurements were collected, of which 68 (88%) were of sufficient quality. With relatively limited exclusion rates (below 12%) in a home setting with regards to ECG measurements, the belt seems to be particularly relevant for home-based patient follow-up investigations.

Further research is still needed, including direct comparisons of alternative measuring approaches for predicting clinical outcomes [26].

## 5. Conclusions

To conclude, our novel wearable chest belt combining ECG and BF provided accurate signals in good agreement with the gold standard PSG. The combination of ECG and BF increases the diagnostic performance compared to either signal alone. Due to its unobtrusive nature, the current wearable device is particularly suitable for multi-night assessments and treatment follow-up at home.

## Figures and Tables

**Table 1:** Patients’ baseline characteristics.

	Characteristics
Number of patients	51
Sex (males/females)	35/16
Age (yr); median [range]	49 [21 to 65]
AHI (/h); median [IQR]	33 (16 to 58)
ODI (/h); median [IQR]	21 (8 to 41)
ESS; median [IQR]	9 (6 to 12)
BMI (kg/m^2^); median [IQR]	31 (IQR: 27 to 35)
SA severe (*n*)	28
SA moderate (*n*)	7
SA mild (*n*)	9
No apnea detected (*n*)	7

## Data Availability

Data are available upon reasonable request to the corresponding author.

## Abbreviations

The following abbreviations are used in this manuscript:

AHI	Apnea-hypopnea index
AUC	Area under the curve
AUC*_loo_*	Area under the curve derived from a leave-one-out cross-validation procedure
BMI	Body mass index
BR	Breathing rate
BRV	Breathing rate variability
BF	Breathing frequency
CSA	Central sleep apnea
MDPI	Multidisciplinary Digital Publishing Institute
ECG	Electrocardiography
EKOS	Ethikkommission Ostschweiz
ESS	Epworth sleepiness scale
FN	False negatives
FP	False positives
HF	High frequency
HR	Heart rate
HRV	Heart rate variability
HRVi	Heart rate variability triangular index
IQR	Inter-quartile rate
IRRR	Inter-quartile range of the RR time series
LF	Low frequency
LFHF	Ratio of low frequency on high frequency
LOOCV	Leave-one-out cross-validation
MADRR	Median of the absolute values of the RR time series
ODI	Oxygen desaturation index
OSA	Obstructive sleep apnea
pNN50	Proportion of interval differences of successive RR intervals greater than 50 ms
PSG	Polysomnography
rMMSD	Root means square of successive differences
ROC	Receiver Operating Characteristics
SA	Sleep apnea
SVM	Support vector machine
TN	True negatives
TP	True positives
ULF	Ultra low frequency
VLF	Very low frequency
